# Differential trajectories of tobacco smoking in people at ultra-high risk for psychosis: Associations with clinical outcomes

**DOI:** 10.3389/fpsyt.2022.869023

**Published:** 2022-07-22

**Authors:** Frederike Schirmbeck, Els van der Ven, Lindy-Lou Boyette, Philip McGuire, Lucia R. Valmaggia, Matthew J. Kempton, Mark van der Gaag, Anita Riecher-Rössler, Neus Barrantes-Vidal, Barnaby Nelson, Marie-Odile Krebs, Stephan Ruhrmann, Gabriele Sachs, Bart P. F. Rutten, Merete Nordentoft, EU-GEI High Risk Study Group, Maria Calem, Lieuwe de Haan, Jentien M. Vermeulen

**Affiliations:** ^1^Department of Psychiatry, Amsterdam UMC, University of Amsterdam, Amsterdam, Netherlands; ^2^Department of Clinical, Neuro- and Developmental Psychology, Vrije Universiteit Amsterdam, Amsterdam, Netherlands; ^3^Department of Clinical Psychology, University of Amsterdam, Amsterdam, Netherlands; ^4^Department of Psychosis Studies, Institute of Psychiatry, Psychology and Neuroscience, King's College London, London, United Kingdom; ^5^Department of Psychology, Institute of Psychiatry, Psychology and Neuroscience, King's College London, London, United Kingdom; ^6^Department of Clinical, Neuro- and Developmental Psychology, Amsterdam Public Mental Health Research Institute, Vrije Universiteit Amsterdam, Amsterdam, Netherlands; ^7^Medical Faculty, University of Basel, Basel, Switzerland; ^8^Departament de Psicologia Clínica i de la Salut, Universitat Autònoma de Barcelona, Barcelona, Spain; ^9^Fundació Sanitària Sant Pere Claver, Spanish Mental Health Research Network (CIBERSAM), Spain; ^10^Orygen, Parkville, VIC, Australia; ^11^Centre for Youth Mental Health, The University of Melbourne, Parkville, VIC, Australia; ^12^University of Paris, GHU-Paris, Sainte-Anne, C'JAAD, Inserm U1266, Institut de Psychiatrie (CNRS 3557), Paris, France; ^13^Department of Psychiatry and Psychotherapy, Faculty of Medicine and University Hospital, University of Cologne, Cologne, Germany; ^14^Department of Psychiatry and Psychotherapy, Medical University of Vienna, Vienna, Austria; ^15^Department of Psychiatry and Neuropsychology, School for Mental Health and Neuroscience, Maastricht University Medical Centre, Maastricht, Netherlands; ^16^Mental Health Center Copenhagen, Department of Clinical Medicine, Copenhagen University Hospital, Copenhagen, Denmark

**Keywords:** ultra-high risk, psychosis, tobacco, smoking, affective symptoms, trajectories

## Abstract

**Objective:**

People at ultra-high risk (UHR) for psychosis have a high prevalence of tobacco smoking, and rates are even higher among the subgroup that later develop a psychotic disorder. However, the longitudinal relationship between the course of tobacco smoking and clinical outcomes in UHR subjects is unknown.

**Methods:**

We investigated associations between tobacco smoking and clinical outcomes in a prospective study of UHR individuals (*n* = 324). Latent class mixed model analyses were used to identify trajectories of smoking severity. Mixed effects models were applied to investigate associations between smoking trajectory class and the course of attenuated psychotic symptoms (APS) and affective symptoms, as assessed using the CAARMS.

**Results:**

We identified four different classes of smoking trajectory: (i) Persistently High (*n* = 110), (ii) Decreasing (*n* = 29), (iii) Persistently Low (*n* = 165) and (iv) Increasing (*n* = 20). At two-year follow-up, there had been a greater increase in APS in the Persistently High class than for both the Persistently Low (ES = 9.77, SE = 4.87, *p* = 0.046) and Decreasing (ES = 18.18, SE = 7.61, *p* = 0.018) classes. There were no differences between smoking classes in the incidence of psychosis. There was a greater reduction in the severity of emotional disturbance and general symptoms in the Decreasing class than in the High (ES = −10.40, SE = 3.41, *p* = 0.003; ES = −22.36, SE = 10.07, *p* = 0.027), Increasing (ES = −11.35, SE = 4.55, *p* = 0.014; ES = −25.58, SE = 13.17, *p* = 0.050) and Low (ES = −11.38, SE = 3.29, *p* = 0.001; ES = −27.55, SE = 9.78, *p* = 0.005) classes, respectively.

**Conclusions:**

These findings suggests that in UHR subjects persistent tobacco smoking is associated with an unfavorable course of psychotic symptoms, whereas decrease in the number of cigarettes smoked is associated with improvement in affective symptoms. Future research into smoking cessation interventions in the early stages of psychoses is required to shine light on the potential of modifying smoking behavior and its relation to clinical outcomes.

## Introduction

The prevalence of tobacco smoking is much higher in patients with psychosis (61.6%) (1), and individuals at ultra-high risk for psychosis (UHR) (up to 53%) (2, 3) than in the general population (25.9%) (4). In addition to an increased risk for somatic morbidity and mortality, tobacco smoking is associated with an increased incidence of psychotic disorders (5, 6) and a higher level of symptoms in patients with a psychotic disorder (7–9). In the general population and UHR samples, some studies have found an association between tobacco smoking and severity of subclinical or attenuated psychotic symptoms (10–12), while other studies have not (2, 3). The cross-sectional nature of most studies and categorical approach on tobacco smoking leaves differences in the severity and course unrecognized. Investigating different long-term trajectories of tobacco smoking and their associations with clinical outcomes in UHR individuals may help to identify subgroups in whom the effects of tobacco smoking may be particularly detrimental and are therefore most suitable for clinical interventions aimed at reducing tobacco use. It is possible that not all tobacco users are equally at risk for psychotic symptom exacerbation but that heavy users or those who increase their use are at higher risk of poor clinical outcomes. In this line, one prospective study from the Northern Finland Birth Cohort 1986 found a greater risk for subsequent psychosis in the heaviest smoking category (13). Regarding symptomatic outcome other than psychotic symptoms, a recent prospective cohort study found specifically early onset and heavy smoking as risk factors for affective symptoms later in life (14). Accordingly, another study in UHR individuals found a larger number of cigarettes smoked per day associated with more severe general symptoms including anxiety and depression (2).

To the best of our knowledge, different prospective patterns of smoking behavior and possible differential associations with symptomatic outcome have not yet been investigated in UHR populations. Applying advanced methods to detect trajectories of tobacco smoking as a possible modifiable risk factor could contribute to the efforts of prevention. We therefore aimed to identify 2-year trajectories of tobacco smoking behavior in UHR individuals who were recruited to the multicenter European Gene-Environment Interactions (EU-GEI) study. Second, we sought to examine sociodemographic and clinical characteristics associated with identified trajectory classes. Finally, we aimed to examine associations between trajectories and the course of attenuated psychotic symptoms (APS), including the risk of transition to psychosis, as well as associations between trajectory class and the course of emotional disturbance and general symptoms as assessed with the Comprehensive Assessment of At-Risk Mental States (CAARMS). We hypothesized that more unfavorable tobacco smoking trajectories would be associated with a more negative course of symptoms and increased risk for transition to psychosis.

## Methods

### Study design and participants

Data were collected as part of EU-GEI study, from May 2010 to April 2015 (15). The study methodology has previously been described in detail elsewhere (16). In short, the study had a naturalistic, prospective design, consisting of a baseline and two or three follow-up assessments, depending on the outcome measure. Subjects were recruited from 11 mental healthcare institutions in London, Amsterdam, The Hague, Vienna, Basel, Cologne, Melbourne, Kortenberg, Paris, Barcelona and São Paulo. The study protocol was approved by the Medical Ethics Committees at each participating sites. EU-GEI was conducted in accordance with the Declaration of Helsinki.

Typical age of participants was 18–35 years but not restricted to due to variation between sites in the age at which persons are accepted by clinical services. Subjects were eligible for the study if they met criteria of the CAARMS (17) for the UHR state classified into one or more of the following three groups: (1) GRD: schizotypal personality disorder or having a first degree relative with a psychotic disorder and experiencing a significant decline in or chronic low psychosocial functioning, (2) APS: having positive psychotic symptoms that do not reach the threshold levels for psychosis (3) BLIPS: an experience of a recent brief psychotic episode which remitted within a week without use of antipsychotic medications. Psychometric features of the UHR state have been described elsewhere (18). Exclusion criteria were an intelligence quotient (IQ) below 60 and the prior experience of a psychotic episode of more than 1 week as assessed by the CAARMS.

### Assessment

Participants were invited for face-to-face follow-up meetings at baseline, and 6 months (limited data as this assessment was introduced later in the course of the study), 12 months and 24 months after baseline. Information regarding transition to psychosis were followed up for 2 years using available clinical records, in case face-to-face meetings were not possible.

Tobacco smoking was assessed with the Composite International Diagnostic Interview (CIDI) (19). The CIDI defines smokers as people who smoked daily during at least 1 month over the past 12 months. In addition, participants were asked how many cigarettes they smoked per day in the time frame they smoked the most during the past months. Studies have confirmed good test-retest and interrater reliability of the CIDI as well as good agreement of CIDI diagnosis with routine clinical diagnosis and applied checklists (20). Sociodemographic and clinical characteristics at baseline included age, gender, ethnicity, education in years, current employment status, IQ and medication use. General functioning was assessed with the disability score of the General Assessment of Functioning Scale (GAF-d) (21). The GAF proved to be a reliable and valid measure of psychiatric disturbances (22, 23). Cannabis use was measured with the Cannabis Experience Questionnaire (CEQ) asking participants whether or not they currently use cannabis. The experience of childhood trauma was assessed with the Childhood Trauma Questionnaire (CTQ) (24) a 25-item self-report questionnaire assessing traumatic events before the age of 17 including emotional abuse, physical abuse, sexual abuse, emotional neglect, and physical neglect. Good reliability and validity of the CTQ has been reported in the general population (25), as well as in patients with psychotic disorders (26).

Attenuated psychotic and affective symptoms were assessed with the CAARMS (17), a semi-structured interview with a total of 27 items, clustered in seven subscales. For the current study the following three subscales were used: APS included items measuring unusual thought content, non-bizarre ideas, perceptual abnormalities and disorganized speech. Emotional disturbance included items measuring subjective emotional disturbance, observed blunted and observed inappropriate affect. General symptoms included symptoms of depression, anxiety, obsessive compulsive disorder, mania, suicidality and self-harm, mood swings, dissociative symptoms and impaired tolerance to normal stress.

Symptom severity was operationalized by summing intensity^*^frequency scores of the corresponding items (27, 28). Good reliability and prognostic validity of the CAARMS has been reported (17). The prospective course of CAARMS positive, emotional disturbance and general symptoms was assessed at baseline, 1 and 2 years follow up, in addition to the risk of transition defined as the development of psychotic disorder according to the CAARMS (29).

#### Covariates

A-priori selected potential confounders based on previous literature (2, 30) including age, gender, socioeconomic status as assessed with education in years and current employment status, childhood trauma, and cannabis use.

### Statistical analysis

Latent class mixed model analysis (LCMM) was used to empirically identify and visualize clusters of participants with similar trajectories of tobacco smoking over time within one sample. For reporting of study design and analyses we followed state-of-the-art guidelines (GRoLTS checklist) (31).

Missing values at baseline were replaced applying multiple imputation procedure to be able to include participants with at least one assessment. With maximum likelihood (ML) estimation LCMM then makes use of all available data, regardless of intermittent missing data and/or later dropout. Subject and time were used to infer latent class trajectories of cigarettes smoked per day. The actual individual time of measurement (days since baseline) was used to account for possible deviation around the planned assessment date. The maximum observational period was set to <1,000 days to avoid including large outlying values (>2SD).

Unconditional LCMM were used to describe the “raw” latent trajectories of smoking without imposing any conditions/predictors on the model. Starting with a one-class model, we fitted models with increasing numbers of classes until we reached the inflection point of the Akaike information criterion (AIC) and Bayesian information criterion (BIC). The AIC can be used to identify the point at which the benefits of improved model fit are outweighed by the cost of the model in terms of its complexity and thus helps to prevent overfitting of the data. In addition, we also examined the somewhat stricter Bayesian information criterion, and the log-likelihood (LL). The latter is a measure of goodness of model fit regardless of model complexity. Finally, posterior probabilities of class membership for each patient were computed using the Bayes theorem (32). According to the GRoLTS checklist the final model was selected based on both statistical (log-likelihood, AIC, BIC) and clinical (class size, distinctness of class-specific trajectories, likelihood of class membership based on posterior probabilities) considerations.

According to the standard Three-Step Method (31), unconditional trajectories were identified as described above (step 1) and class membership was saved and merged with the original data (step 2). To examine associations between baseline characteristics with most likely trajectory class membership chi-square test and analyses of variance (ANOVA) were conducted for categorical and continuous variables, respectively (step 3).

To examine associations between longitudinal outcome in APS and affective symptoms in relation to trajectories of smoking, mixed effects models were applied. The model included fixed effects for time (as categorical), most likely class membership (based on the LCMM as reported above), their two-way interaction, a random intercept and an autoregressive error covariance structure to account for within-subject correlation over time. Pre-specified contrasts were tested from the model with the low and decreasing trajectory class as reference for sequential follow-up assessments. Analyses were controlled for a priori selected covariates.

Associations between trajectory class and risk to transition to psychotic disorders within the 2-year follow-up interval was assessed using Cox proportional hazard regression analyses after assessing the proportional hazards assumption. The overall cumulative risk of psychosis onset for individuals with different trajectories was plotted with the Kaplan–Meier cumulative event function and 95% confidence intervals (CI) (33).

LCMM was conducted using the lcmm R package (34), cox proportional hazard regression analyses were analyzed using survival R package (35) and survminer R package (33) to plot Kaplan–Meier functions with R version 3.6.2. All other analyses were performed using SPSS version 26.

## Results

### Sample characteristics

Of the 345 CHR-P individuals participating in EU-GEI, 324 provided data on the number of cigarettes smoked per day. Of these 324 individuals, 39 (12.0%) were assessed with the CIDI and CAARMS at 6-months follow-up, 174 (53.7%) at 1 year and 127 (39.2%) at 2 years follow-up, respectively. Median follow-up period in days was 196 (range 21–272) for 6 months, 380 days (range 187–580) for 1-year, and 757 days (min = 535 and max = 993) for 2-year assessments. See flow-chart Supplementary Figure 1.

Data regarding missingness at baseline, and comparisons between dropouts and completers at 1-year are presented as Supplementary Sections 2, 3. Comparing completers and dropouts at 1-year follow-up showed no significant differences in number of cigarettes smoked per day, age, gender, current employment, GAF disability scores, experienced childhood trauma and current cannabis use at baseline. Dropouts had a lower IQ (*t* = 3.380, *p* = 0.001), less years of education (*t* = 4.057, *p* < 0.001) and were more likely to have an ethnic minority background (X = 6.521, *p* = 0.011).

Overall, 13 (4.0%) of the 324 participants who were included in our study completed all four assessments, 103 (31.8%) three, 95 (29.3%) two and 113 (34.9%) one assessment. Attrition within the analysis sample seemed mostly at random as the number of assessments was not associated with tobacco smoking, CAARMS outcome, trajectory class membership, gender, ethnicity, current employment, cannabis use, GAF, trauma. Participants with one or two assessments were significantly younger compared to those who completed three assessments.

### Trajectories of smoking behavior

A 4-class model was selected for smoking trajectories as the associated BIC was the lowest among the tested models (see Table 1). For this 4-class model, mean class probabilities were moderate to high (0.78- 0.95), suggesting individuals had a 78–95% probability to be correctly assigned to one of the four latent classes.

**Table 1:** Model Fit Parameters for LCMM of numbers of cigarettes smoked with One to Five Classes.

**Number of classes**	**Number of parameters**	**AIC**	**BIC**	**Max log-likelihood**	**Posterior probability**	**Sample size per class**
1	11	3794.156	3835.744	−1886.078		
2	14	3618.716	3671.647	−1795.358	0.98	137 / 187
3	17	3585.048	3649.321	−1775.524	0.85–0.98	43 / 173 / 108
4	20	3528.232	3603.847	−1744.116	0.78–0.95	110 / 29 / 165 / 20
5	23	3573.718	3660.675	−1763.859	0.59–0.92	43 / 0 / 11 / 173 / 97

*AIC, Akaike Information Criterion; BIC, Bayesian Information Criterion; LCMM, Latent Class MixedModeling.*

*The bold values indicate the statistically significant results (p ≥ 0.05)*.

**Figure 1:**
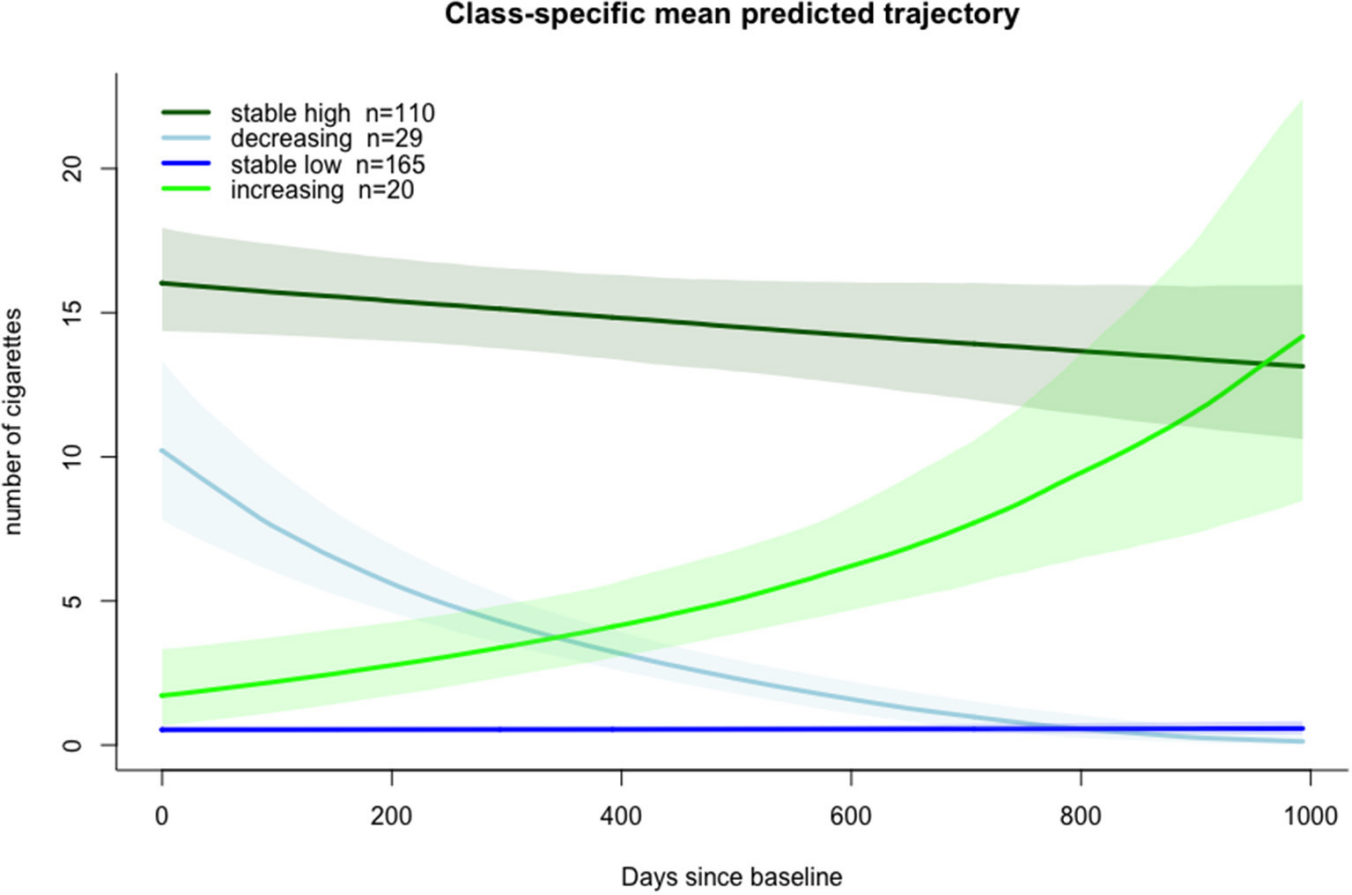
Model estimated class-specific mean predicted trajectories of tobacco smoking with 95% confidence intervals.

After visual inspection of the identified trajectories, the smoking classes were labeled as: (i) Persistently High (*n* = 110), (ii) Decreasing (*n* = 29), (iii) Persistently Low (*n* = 165) and (iv) Increasing (*n* = 20), see Figure 1. Individuals in the Persistently High smoking trajectory class smoked on average 15.23 (SD = 8.34) cigarettes per day across time points, patients in the Low smoking trajectory class smoked no cigarettes or a consistently low number (mean of 0.24 (0.84) and maximum of 5 cigarettes per day). For observed individual courses of cigarettes smoked per day by most likely trajectory membership see Supplementary Figures 2A–D.

### Trajectory class membership and baseline characteristics

Comparisons between trajectory classes on baseline characteristics are presented in Table 2. Classes did not significantly differ in gender, ethnicity, years of education, GAF disability scores, IQ score or medication use. The Persistently High and Increasing class was older compared to the Persistently Low smoking class. In term of cannabis use, subjects in the Low smoking class reported less current cannabis use compared to all other trajectory classes.

**Table 2:** Baseline information on sociodemographic and clinical variables by trajectory class.

	**Class 1** **(persistent high)** ***N*** = **110**	**Class 2** **(decreasing)** ***N*** = **29**	**Class 3** **(persistent low)** ***N*** = **165**	**Class 4** **(increasing)** ***N*** = **20**	**Group** **comparisons**	**Pairwise** **comparisons**
Age	23.17 (5.22)	22.00 (5.69)	21.74 (4.61)	24.35 (5.09)	*F* = 2.964, *p* = 0.032	High > Low, Inc > Low
Gender (% male)	58.2	55.2	48.5	65.0	X = 3.731, *p* = 0.292	
Ethnicity (% white)	73.6	65.5	70.9	60.0	X = 1.94, *p* = 0.585	
Years of education	13.98 (3.19)	13.93 (2.00)	14.42 (3.18)	15.40 (2.60)	*F* = 1.485, *p* = 0.219	
Now paid work or student ( % yes)	49.1	51.7	64.0	70.0	X = 7.631, *p* = 0.054	
GAF disability	53.83 (10.90)	55.07 (11.75)	57.19 (13.50)	53.90 (12.34)	*F* = 1.781, *p* = 0.151	
Cannabis use (% yes)	39.1	37.9	12.7	35.0	X = 28.308, *p* <0.001	High > Low, Inc > Low, Dec > Low
Childhood trauma	9.73 (3.09)	10.87 (3.58)	9.71 (3.11)	9.67 (3.14)	*F* = 1.840, *p* = 0.146	
IQ	95.94 (16.01)	96.22 (19.79)	100.66 (17.40)	99.89 (14.57)	*F* = 2.098, *p* = 0.101	
Medication*						
Antidepressants/ Mood stabilizers	26 (28.6)	5 (20.8)	43 (31.9)	6 (37.5)	X = 1.695, *p* = 0.638	
Anxiolytics	11 (12.1)	0	13 (9.6)	2 (12.5)	X = 3.290, *p* = 0.349	
Antipsychotics	11 (12.1)	1 (4.2)	12 (8.9)	3 (18.8)	X = 2.850, *p* = 0.415	

SD, standard deviation; Cigs/day, number of cigarettes per day; GAF, global assessment of functioning. *Information from a subsample of n = 266.

*The bold values indicate the statistically significant results (p ≥ 0.05)*.

### Prospective outcome associated with trajectory class

As only a small subgroup of participants provided CAARMS data at 6 months follow-up (see flow-chart, Supplement Figure 1), we only included baseline, 1 and 2-years follow-up data of APS, emotional disturbance and general symptoms in the analyses on smoking trajectory and clinical outcomes.

Although the overall trajectory class by time interaction effect for APS was not significant (*F* = 1.677, *p* = 0.127), pre-specified contrasts with the Low and Decreasing trajectory classes as reference, revealed a significant increase in APS in the High trajectory class compared to the Low trajectory class (ES = 9.770, SE = 4.873, *p* = 0.046) and Decreasing trajectory class (ES = 18.182, SE = 7.612, *p* = 0.018) at 2-years follow-up, respectively (Table 3). A significant overall interaction effect was found for CAARMS emotional disturbance (*F* = 2.308 *p* = 0.035). Pre-specified contrasts showed more decrease in the Decreasing trajectory class at 2 years compared with the High (ES = −10.396, SE = 3.414, *p* = 0.003), Increasing (ES = −11.347, SE = 4.551, *p* = 0.014) and Low class (ES = −11.378, SE = 3.290, *p* = 0.001) (Table 4). No significant overall interaction effect was found for CAARMS general symptoms (*F* = 1.494 *p* = 0.180). Pre-specified contrasts showed more decrease in the Decreasing trajectory group at 2 years compared with the High (ES = −22.356, SE = 10.074, *p* = 0.027), Increasing (ES = −25.582, SE = 13.169, *p* = 0.050) and Low smoking class (ES = −27.553, SE = 9.783, *p* = 0.005) (Table 5).

**Table 3:** Results of mixed model analyses of the effect of trajectory class membership on attenuated positive symptoms (APS).

**Outcome**	**Fixed effects**		**Estimate**	**SE**	***p*** **-value**	**95% CI**	
**APS**	Reference low smokingclass						
		Intercept	30.574	7.541	0.000	15.735	45.412
	Trajectoryclass	High	−3.329	2.764	0.229	−8.760	2.102
		Decreasing	7.835	4.349	0.072	−0.710	16.381
		Increasing	1.157	5.215	0.824	−9.088	11.403
	Time	1 year	−12.691	2.616	0.000	−17.838	−7.542
		2 years	−17.189	3.135	0.000	−23.358	−11.019
	Class*Time	High*1 year	−0.092	3.949	0.981	−7.865	7.679
		High*2 years	9.770	4.873	0.046	0.179	19.360
		Decreasing*1 year	1.508	5.849	0.797	−10.004	13.021
		Decreasing*2 years	−8.412	7.328	0.252	−22.838	6.014
		Increasing*1 year	3.652	6.877	0.596	−9.887	17.191
		Increasing*2 years	10.324	8.132	0.206	−5.697	26.345
	Reference decreasingclass						
		Intercept	38.409	8.402	0.000	21.882	54.936
	Trajectoryclass	High	−11.164	4.46	0.013	−19.935	−2.393
		Low	−7.835	4.349	0.072	−16.381	0.710
		Increasing	−6.678	6.300	0.290	−19.056	5.699
	Time	1 year	−11.181	5.232	0.033	−21.481	−0.881
		2 years	−25.601	6.627	0.000	−38.650	−12.552
	Class*Time	High*1 year	−1.601	6.013	0.790	−13.437	10.234
		High*2 years	18.182	7.612	0.018	3.196	33.168
		Low*1year	−1.508	5.849	0.797	−13.021	10.004
		Low *2 years	8.412	7.328	0.252	−6.014	22.838
		Increasing*1 year	2.143	8.233	0.795	−14.065	18.352
		Increasing*2 years	18.736	10.012	0.062	−0.985	38.457

*The bold values indicate the statistically significant results (p ≥ 0.05)*.

**Table 4:** Results of mixed model analyses of the effect of trajectory class membership on emotional disturbances.

**Outcome**	**Fixed effects**	**Parameter**	**Estimate**	**SE**	***p*** **-value**	**95% CI**	
**Emotional**	Reference low smoking class						
		Intercept	7,123	3,640	0.051	−0.044	14,290
	Trajectoryclass	High	1,333	1,303	0.307	−1,228	3,895
		Decreasing	7,653	2,047	0.000	3,629	11,677
		Increasing	−1,821	2,456	0.459	−6,648	3,006
	Time	1 year	−5,923	1,388	0.000	−8,660	−3,186
		2 years	−5,405	1,442	0.000	−8,254	−2,555
	Class*Time	High*1 year	1,906	2,094	0.364	−2,220	6,033
		High*2 years	−,982	2,228	0.660	−5,385	3,420
		Decreasing*1 year	−1,265	3,240	0.697	−7,653	5,123
		Decreasing*2 years	−11,378	3,290	0.001	−17,883	−4,873
		Increasing*1 year	0.063	3,556	0.986	−6,951	7,077
		Increasing*2 years	−,031	3,745	0.993	−7,440	7,378
	Reference decreasing class						
		Intercept	14,776	4,034	0.000	6,837	22,716
	Trajectory class	High	−6,320	2,101	0.003	−10,449	−2,191
		Low	−7,653	2,047	0.000	−11,677	−3,629
		Increasing	−9,474	2,966	0.001	−15,304	−3,645
		1 year	−7,188	2,927	0.015	−12,960	−1,416
		2 years	−16,783	2,958	0.000	−22,633	−10,932
		High*1 year	3,171	3,324	0.341	−3,382	9,725
		High*2 years	10,396	3,414	0.003	3,645	17,146
		Low*1year	1,265	3,240	0.697	−5,123	7,653
		Low *2 years	11,378	3,290	0.001	4,873	17,883
		Increasing*1 year	1,328	4,394	0.763	−7,337	9,993
		Increasing*2 years	11,347	4,550	0.014	2,345	20,348

*The bold values indicate the statistically significant results (p ≥ 0.05)*.

**Table 5:** Results of mixed model analyses of the effect of trajectory class membership on general symptoms.

**Outcome**	**Fixed effects**	**Parameter**	**Estimate**	**SE**	***p*** **-value**	**95% CI**	
**General**	**Reference low smoking class**						
		Intercept	25,909	10,202	,012	5,833	45,984
	Trajectoryclass	High	5,588	3,658	,127	−1,602	12,777
		Decreasing	8,787	5,749	,127	−2,510	20,085
		Increasing	5,943	6,894	,389	−7,605	19,489
	Time	1 year	−17,557	3,404	,000	−24,268	−10,866
		2 years	−14,768	4,136	,000	−22,893	−6,642
	Class*Time	High*1 year	0,219	5,094	,966	−9,810	10,248
		High*2 years	−5,197	6,304	,410	−17,584	7,189
		Decreasing*1 year	−4,602	7,767	,554	−19,896	10,690
		Decreasing*2 years	−27,553	9,784	,005	−46,778	−8,329
		Increasing*1 year	6,197	8,469	,465	−10,481	22,876
		Increasing*2 years	−1,971	10,575	,852	−22,753	18,810
	**Reference decreasing class**						
		Intercept	34,697	11,314	,002	12,440	56,953
	Trajectory class	High	−3,199	5,897	,588	−14,787	8,388
		Low	−8,787	5,749	,127	−20,085	2,510
		Increasing	−2,845	8,325	,733	−19,204	13,514
		1 year	−22,169	6,956	,002	−35,927	−8,412
		2 years	−42,321	8,869	,000	−59,748	−24,893
		High*1 year	4,821	7,952	,545	−10,837	20,479
		High*2 years	22,356	10,075	,027	2,560	42,152
		Low*1year	4,602	7,767	,554	−10,691	19,896
		Low *2 years	27,553	9,78	,005	8,32	46,778
		Increasing*1 year	10,799	10,437	,302	−9,756	31,356
		Increasing*2 years	25,582	13,169	,050	−,296	51,460

*The bold values indicate the statistically significant results (p ≥ 0.05)*.

**Figure 2:**
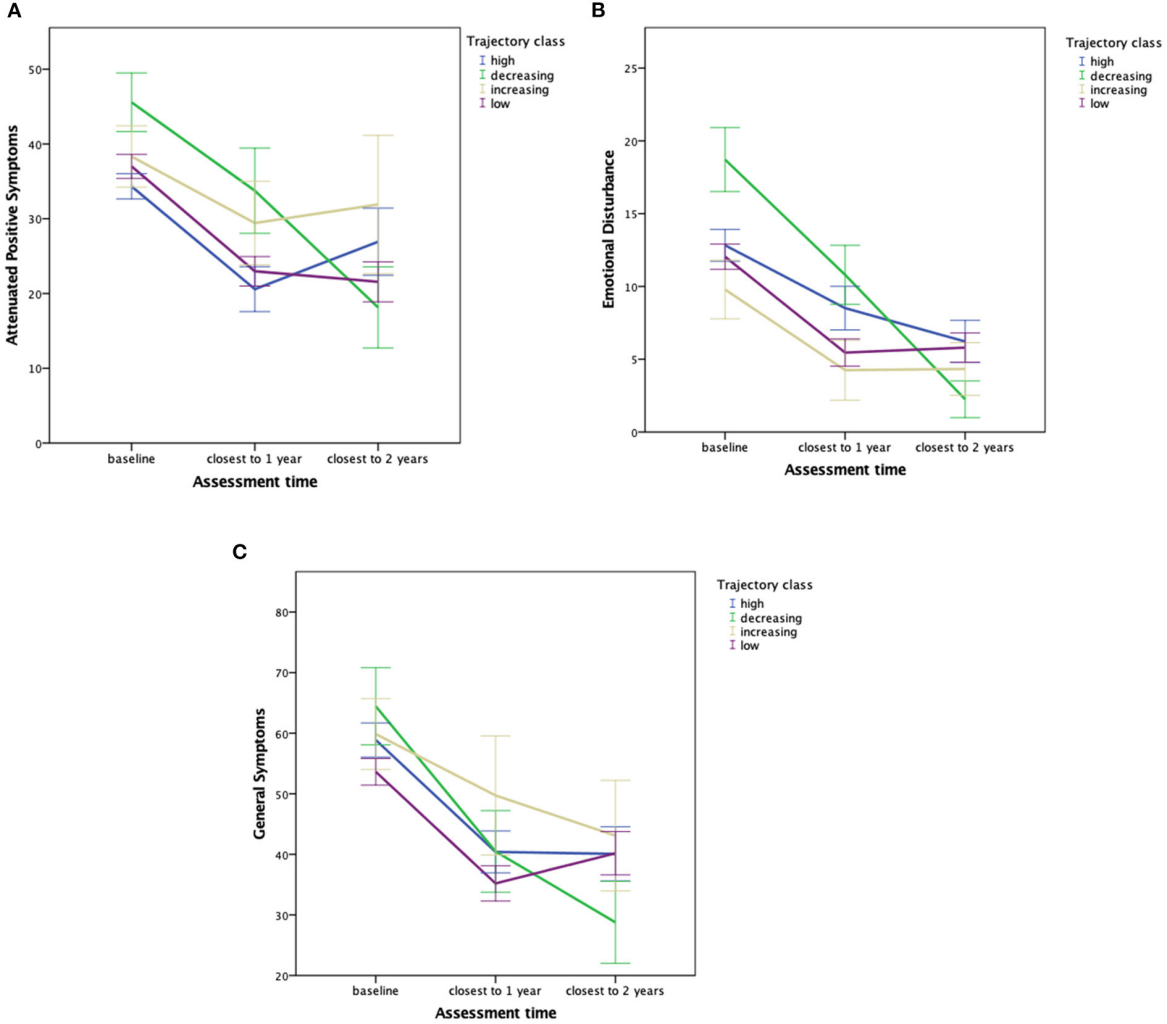
**(A)** Model estimated means and 1-standard errors of APS scores predicted by most likely trajectory class membership and assessment time. **(B)** Model estimated means and 1-standard errors of emotional disturbance scores predicted by most likely trajectory class membership and assessment time. **(C)** Model estimated means and 1-standard errors of general symptom scores predicted by most likely trajectory class membership and assessment time.

Model estimated means for CAARMS APS, emotional disturbance and general symptoms by trajectory class are presented in Figures 2A–C, respectively.

#### Transition

**Figure 3:**
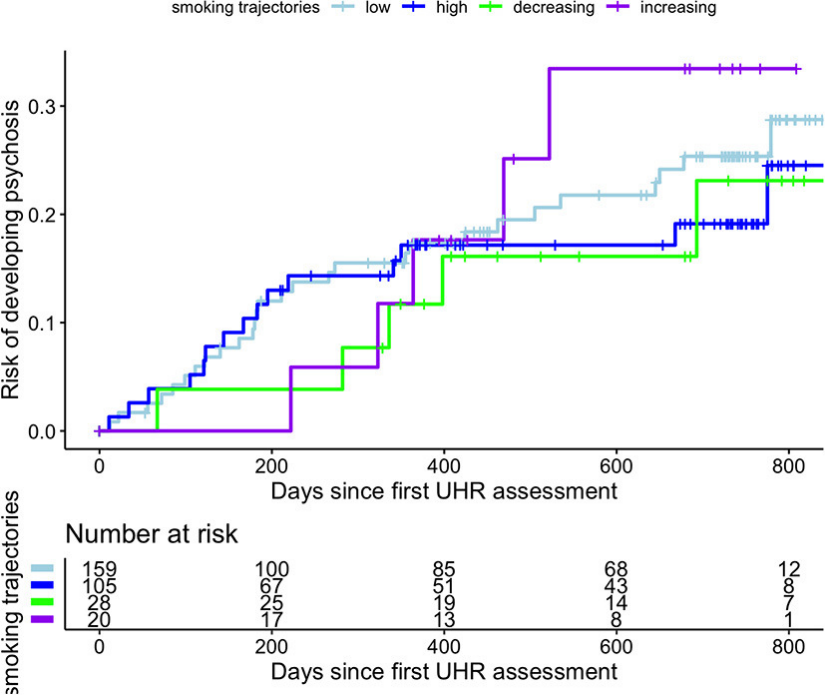
Cumulative event Kaplan–Meier function for risk of development of psychotic disorders in 312 ultra-high risk (UHR) individuals stratified for smoking trajectory class.

Transition to psychosis data within the 1,000 days' timeframe was available in 312 participants of the current sample, who were assigned to one of the four smoking trajectory classes. Within the 2-year period, 53 (16.8%) UHR individuals transitioned to psychosis. Transition occurred in 15 (14.3%) individuals from the Persistently High smoking class, 5 (25.0%) from the Increasing, 5 (17.8%) from the Decreasing and 28 (17.6%) form the Persistently Low class. The median time to transition was 220.5 days (25th−75^th^ percentiles 122–398). The last transition was observed at 779 days when 28 individuals were still at-risk. Cox proportional hazard regression analyses showed no increased cumulative risk to develop a psychotic disorder in the High HR = 0.84 (95%CI:0.45–1.6, *p* = 0.593), Decreasing HR = 0.75 (95%CI:0.29–1.9, *p* = 0.556) or Increasing 1.25 (95%CI:0.48–3.3, *p* = 0.647) trajectory class compared to the Low class, while again controlling a priori defined covariates. The corresponding Kaplan–Meier cumulative risk of psychosis curves are depicted in Figure 3.

## Discussion

To our knowledge, this is the first study investigating differential trajectories of tobacco smoking in UHR individuals. Our findings show a clustering around four distinct trajectory classes, with the majority of participants (84%) reporting either persistently high (34%) or persistently low (51%) tobacco smoking across the 2 year assessment period. Smaller subgroups showed a longitudinal decrease (9%) or an increase (6%) in number of cigarettes smoked. The High and Increasing trajectory class was older and reported more cannabis use when compared to the Low trajectory class. Identified trajectory classes did not significantly differ on any other sociodemographic or clinical characteristics at baseline.

Regarding associations between trajectory class membership and the course of symptoms, a persistently high level of tobacco smoking was associated with an unfavorable course of APS severity at 2-years follow up: in contrast the Persistently Low and Decreasing trajectory classes showed a continuous decrease in APS severity over time, the Persistently High smoking class showed increasing severity at 2-years follow-up (see Figure 2A). Although no increased risk for transition was found in the Persistently High or Increasing smoking trajectory class, interpretation of this finding is limited by the small transition numbers per class. Furthermore, results show a larger decrease in emotional disturbance and general symptoms in the Decreasing trajectory class compared to all other classes (see Figures 2B,C).

Noteworthy, we can only compare our results with studies conducted in psychiatric patients or the general population, which limits comparability. In line with our finding of a larger reduction of emotional symptoms in the decreasing smoking class, a previous general population study found smoking cessation associated with a decrease in depressive symptoms and increased resilience over a two-year period (36). A recent meta-analysis showed that smoking discontinuation led to an improvement of mental health symptoms, also in psychiatric patients (37). Although our results suggest an unfavorable course of APS severity at the last assessment in the Persistently High smoking class, we did not find an increased risk for transition, as has previously been reported for the heaviest smoking category in The Northern Finland Birth Cohort 1986 study (13).

Regarding associations between baseline characteristics and smoking trajectory class membership, associations with age and cannabis use are in line with earlier research in the general population (38, 39). A prospective investigation of first episode psychosis patients found cannabis use to be associated with lower smoking cessation rates, specifically in female smokers (40). We also found higher cannabis use in the Persistently High and Increasing class, however not in the Decreasing class compared to the Low smoking trajectory class. Due to small samples sizes we were unable to investigate possible moderating effects of gender.

A previous study showed cannabis use to be a possible mediating factor between adolescent smoking trajectory and adult mental health (14). In another study, authors directly compared the effect of patterns of cigarette and cannabis use on subsequent psychotic experiences in a prospective cohort study and found an almost 2-fold increased risk in early-onset cigarette-only users and an almost 4-fold increased risk in early-onset cannabis users, compared with non-users (41). In contrast to a previous study, we did not find childhood trauma to be associated with an unfavorable smoking course. Yoon et al. found that adolescents with early childhood trauma were 2 to 3 times more likely to show increase in smoking behavior compared to the persistently low smoking trajectory class (42).

Different non-mutually exclusive mechanisms have been proposed to explain the link between tobacco smoking and mental health symptoms, including biological explanations such as nicotine-induced elevated dopamine release (6, 43) and shared genetic vulnerability (44). On the behavioral level, both maladaptive coping and misattribution are thought to play a key role in the relationship between smoking and symptoms. Smoking may represent a maladaptive strategy of trying to cope with the stress of experienced symptoms, potentially resulting into even higher levels of symptoms (45). Smokers may misattribute the relief of withdrawal symptoms such as irritability, anxiety, and depression after smoking to the perception that smoking has psychological benefits, which also makes them less likely to stop smoking (46). A growing body of evidence showed that smoking is not effective to alleviate symptoms but stopping smoking is associated with improvement of mental health in both the general population as clinical samples, arguing against the self-medication hypothesis (7, 37, 47). So far, most research suggests a bidirectional relation between smoking and symptoms. Experienced stress and related emotional distress may heighten the risk of smoking initiation, progression, maintenance, cessation avoidance, and relapse (48). Conversely, smoking and associated withdrawal symptoms cause stress and emotional disturbances. Lastly, those with more severe symptoms might have difficulties in stopping smoking or decreasing the number of cigarettes smoked per day (49, 50).

## Limitations

Our results should be interpreted in the light of several limitations. First, from a temporality point of view there was no information available on whether smoking initiation took place before or after the occurrence of first psychotic experiences, precluding causal interpretations. A large cohort study investigating longitudinal classes of tobacco use in minors showed that specifically early-onset tobacco use was correlated with subsequent onset of psychotic experiences (41). In order to determine causal interrelations between tobacco smoking and the course of symptoms, future studies should seek to assess tobacco smoking in the daily life of UHR individuals. This would allow the investigation of moment-to-moment associations between smoking behavior and psychotic or affective experiences. Second, the relatively small number of individuals assigned to the increasing and decreasing trajectory class, in combination with considerable loss to follow-up during the course of the study, limits the reliability of the assessed associations between identified trajectories and prospective outcome. Although sensitivity analyses (see Supplementary Section 5) resulted in comparable tobacco smoking trajectory classes, careful interpretation is warranted and there is a need for replication with prospective data of a larger sample. Third, loss to follow-up might further have influenced our findings as dropouts showed lower IQ, less years of education and were more likely to have an ethnic minority background compared to completers. These differences limit the generalizability of findings. Fourth, generalizability is also limited to help-seeking UHR individuals. Fifth, information on other potential confounders affecting the course of psychopathology such as the effect of medication use was only available in a subgroup of participants and therefore not included in the analyses. In the subgroup with known medication status, no significant differences between identified trajectory class membership were found (see Table 2). Unfortunately, information on psychological interventions during the course of the study was not available at all sites. To account for between-trajectory differences in cannabis use we controlled for this variable in subsequent analyses, however possible interacting effects of these substances on clinical outcome are worth investigating in larger samples in the future.

## Conclusion and clinical implications

Findings showed interrelations between a persistently high level of tobacco use and an unfavorable course of APS severity and a positive interrelation between reduction in tobacco use and an improvement in affective symptoms over time. More research is needed to understand possible covariation and causal interactions. Although a causal direction cannot be established and bidirectional interrelations are most probable in the current study, smoking cessation interventions in this vulnerable group should receive more attention. UHR individuals experience less intense and frequent symptoms than individuals with established psychosis and it might be easier in this phase to quit smoking. Early intervention smoking cessation programs should therefore be offered when UHR individuals present to psychiatric services. Current findings suggest that differentiating UHR individuals based on patterns of smoking behavior might contribute to identifying subgroups with a higher risk for an unfavorable outcome.

## Data availability statement

The original contributions presented in the study are included in the article/Supplementary Materials, further inquiries can be directed to the corresponding author.

## Ethics statement

The studies involving human participants were reviewed and approved by Local Medisch Ethische Toetsingscommissie (METC), University Medical Center Amsterdam, Location AMC, Amsterdam, the Netherlands (NL32721.018.10). The patients/participants provided their written informed consent to participate in this study.

## EU-GEI high risk study group

Maria Calem^1^, Stefania Tognin^1^, Gemma Modinos^1^, Sara Pisani^1^, Emily P. Hedges^1^, Eva Velthorst^2, 3^, Tamar C. Kraan^2^, Daniella S. van Dam^2^, Nadine Burger^4^, Athena Politis^5^, Joanne Goodall^5^, Stefan Borgwardt^6^, Erich Studerus^6^, Ary Gadelha^7^, Elisa Brietzke^8^, Graccielle Asevedo^7^, Elson Asevedo^7^, Andre Zugman^7^, Tecelli Domínguez-Martínez^9^, Manel Monsonet^10^, Lidia Hinojosa^10^, Paula Cristóbal^10^, Thomas R. Kwapil^11^, Mathilde Kazes^12^, Claire Daban^12^, Julie Bourgin^12^, Olivier Gay^12^, Célia Mam-Lam-Fook^12^, Dorte Nordholm^13^, Lasse Randers^13^, Kristine Krakauer^13^, Louise Birkedal Glenthøj^13^, Dominika Gebhard^14^, Julia Arnhold^15^, Joachim Klosterkötter^14^, Iris Lasser^16^, Bernadette Winklbaur^16^, Philippe A. Delespaul^17, 18^.

^1^Department of Psychosis Studies, Institute of Psychiatry, Psychology and Neuroscience, King's College London, London, United Kingdom; ^2^Department of Psychiatry, Amsterdam UMC, University of Amsterdam, Amsterdam, Netherlands; ^3^Icahn School of Medicine at Mount Sinai, department of Psychiatry, New York, NY, United States; ^4^Parnassia Group, Psychosis Research Institute, The Hague, Netherlands; ^5^Orygen, Parkville, VIC, Australia. ^6^Medical Faculty, University of Basel, Basel, Switzerland; ^7^Depto Psiquiatria, Escola Paulista de Medicina, LiNC-Lab Interdisciplinar Neuroci ncias Cl nicas, Universidade Federal de São Paulo (UNIFESP), São Paulo, Brazil; ^8^Depto Psiquiatria, Escola Paulista de Medicina, Universidade Federal de São Paulo–UNIFESP, Brazil; ^9^CONACYT-Dirección de Investigaciones Epidemiológicas y Psicosociales, Instituto Nacional de Psiquiatría Ramón de la Fuente Muñiz, México, Mexico; ^10^Departament de Psicologia Clínica i de la Salut, Universitat Autònoma de Barcelona, Barcelona, Spain; ^11^Department of Psychology, University of Illinois at Urbana-Champaign, United States; ^12^University Paris Descartes, Hôpital Sainte-Anne, C'JAAD, Service Hospitalo-Universitaire, Inserm U894, Institut de Psychiatrie (CNRS 3557) Paris, France; ^13^Mental Health Center Copenhagen and Center for Clinical Intervention and Neuropsychiatric Schizophrenia Research, CINS, Mental Health Center Glostrup, Mental Health Services in the Capital Region of Copenhagen, University of Copenhagen, Copenhagen, Denmark; ^14^Department of Psychiatry and Psychotherapy, Faculty of Medicine and University Hospital, University of Cologne, Cologne, Germany; ^15^Psyberlin, Berlin, Germany; ^16^Department of Psychiatry and Psychotherapy, Medical University of Vienna, Vienna, Austria; ^17^Department of Psychiatry and Neuropsychology, School for Mental Health and Neuroscience, Maastricht University Medical Centre, Maastricht, Netherlands; ^18^Mondriaan Mental Health Trust, Heerlen, Netherlands.

## Author contributions

FS and JV: literature search, figures, data analysis, data interpretation, and writing. EV and L-LB: literature search, figures, data interpretation, and writing. PM, LV, MK, MG, AR-R, NB-V, BN, M-OK, SR, GS, BR, MN, and LH: conception and design of the cohort study and critical feedback. All authors contributed to the article and approved the submitted version.

## Funding

The European Network of National Schizophrenia Networks Studying Gene-Environment Interactions (EU-GEI) Project was funded by grant agreement HEALTH-F2-2010-241909 (Project EU-GEI) from the European Community's Seventh Framework Programme. Additional support was provided by a Medical Research Council Fellowship to M Kempton (grant MR/J008915/1). BN was supported by an NHMRC Senior Research Fellowship (1137687).

## Conflict of interest

The authors declare that the research was conducted in the absence of any commercial or financial relationships that could be construed as a potential conflict of interest.

## Publisher's note

All claims expressed in this article are solely those of the authors and do not necessarily represent those of their affiliated organizations, or those of the publisher, the editors and the reviewers. Any product that may be evaluated in this article, or claim that may be made by its manufacturer, is not guaranteed or endorsed by the publisher.
