# Supplementary material for: Differential trajectories of tobacco smoking in people at ultra-high risk for psychosis: Associations with clinical outcomes
Source: Front Psychiatry. 2022 Jul 22;13:869023. doi: 10.3389/fpsyt.2022.869023 (PMC9356251; doi:10.3389/fpsyt.2022.869023)

**Supplements**

**Figure 1**. Flowchart of included participants

## Enrollment

## One-year

## Follow-Up

Included in EU-GEI

345 UHR individuals

## Current study

324 UHR individuals with data on both CIDI (324) &

CAARMS (330)

## Six months

## Follow-Up

39 UHR individuals with

data on CIDI (41) &

CAARMS (72)

(this assessment was not conducted at all sites)

g

## Two years

## Follow-Up

174 UHR individuals with

data on CIDI (176) &

CAARMS (178)

(this assessment was not conducted at all sites)

w

127 UHR individuals with

data on CIDI (128) &

CAARMS (132)

(this assessment was not conducted at all sites)

*Abbreviation:* CAARMS: the Comprehensive Assessment of At-Risk Mental States; CIDI: Composite International Diagnostic Interview, UHR: ultra-high risk

**2. Missingness at baseline**

Analyses of missing data at baseline showed a small fraction of missing values on relevant items of the CAARMS, SCID and covariates (0-12.4%) in the sample of 331 individuals (see table 1). Pattern analyses and Little’s MCAR test showed that data were missing completely at random (X=137.385, p=.403).

**Table 1: Number of missing values on assessed variables at baseline**

|  | N | Missing | |
| --- | --- | --- | --- |
|  |  | Count | Percent |
| Age | 324 | 0 | ,0 |
| Gender | 324 | 0 | ,0 |
| Ethnicity  Years of education | 324  324 | 0  0 | ,0  ,0 |
| GAF disability | 320 | 4 | 1,2 |
| Current cannabis use  Childhood trauma questionnaire | 324  321 | 0  3 | ,0  ,9 |
| Now paid work | 310 | 14 | 4,3 |

**3. Table 2: Comparison of dropouts and completers at one-year follow-up on baseline characteristics**

|  | Completer  (n=174) | Dropout  (n=150) | Group comparison |
| --- | --- | --- | --- |
| # of cigarettes smoked | 7.04 (9.33) | 5.62 (8.44) | T=1.423 p=.156 |
| Current cannabis use (%yes) | 46 (26.4) | 36 (24.0) | X=.138, p=.710 |
| Age | 22.88 (4.88) | 21.82 (5.10) | T=1.911 p=.057 |
| Gender (% male) | 99 (56.9%) | 74 (49.3%) | X=.819, p=.366 |
| Ethnicity (% white) | 133 (76.3%) | 93 (62.0%) | X=6.521, p=.011 |
| Now paid work or student ( % yes) | 104 (60.5%) | 71 (52.2%) | X=2.112, p=.146 |
| Years of education | 14.89 (2.903) | 13.53 (3.12) | T=4.057 , p<.001 |
| IQ | 101.16 (16.32) | 94.50 (17.44) | T=3.380, p=.001 |
| GAF | 55.93 (11.65) | 54.86 (13.09) | T=.766 p=.444 |
| Childhood trauma | 9.52 (2.86) | 9.86 (3.46) | T=-.948, p=.344 |

**4. Figure 2a-d:** Observed individual course of numbers of cigarettes smoked per day by most likely trajectory class membership with an overlay of observed means for the a) persistently high, b) decreasing, c) increasing and d) persistently low smoking trajectory class

a)


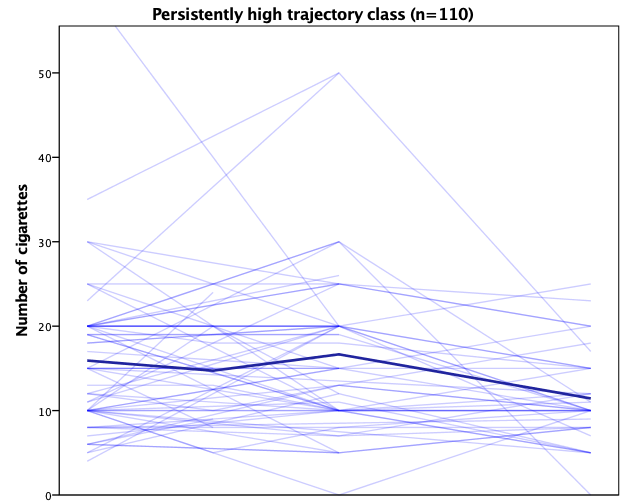


Baseline 6-months 1-year 2 years

N=110 11 59 41

b)


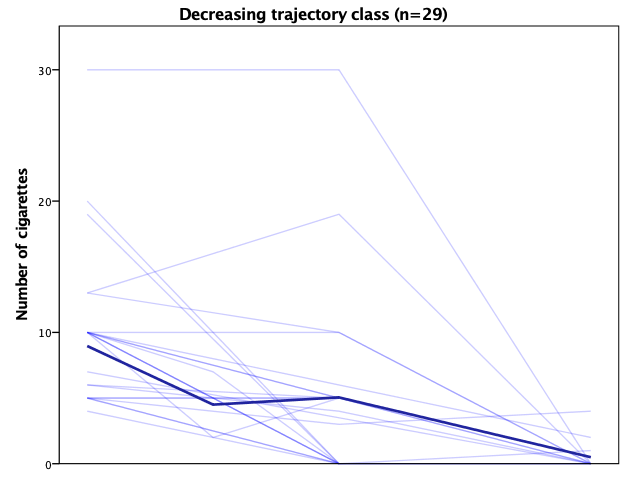


Baseline 6-months 1-year 2 years

N=29 2 19 14

**c)**


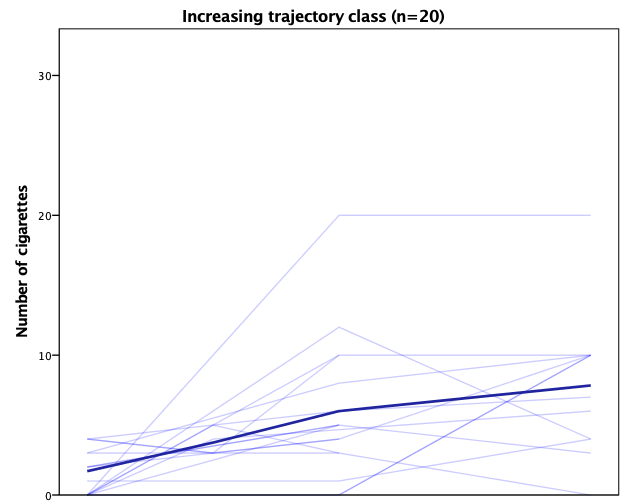


Baseline 6-months 1-year 2 years

N=20 4 16 12

**d)**


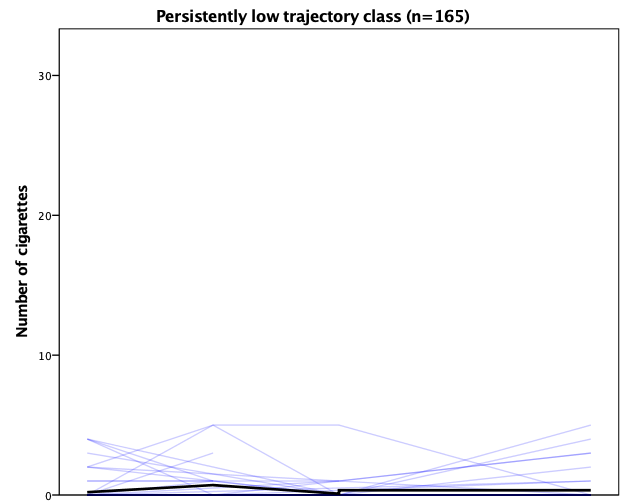


Baseline 6-months 1-year 2 years

N=165 24 73 58

**5. Sensitivity analysis:**

**Table3:** Model Fit Parameters for Latent class mixed model conducted with 211 participants who provided at least one follow-up assessment of numbers of cigarettes smoked with One to Five Classes.

| Number of classes | Number of Parameters | AIC | BIC | Max log-likelihood | Posterior probability | Sample size per class |
| --- | --- | --- | --- | --- | --- | --- |
| 1 | 11 | 3079.983 | 3116.853 | -1528.991 |  |  |
| 2 | 14 | 2989.108 | 3036.034 | -1480.554 | .95-.98 | 87/ 124 |
| 3 | 17 | 2947.581 | 3004.563 | -1456.791 | .89-.98 | 80 / 19 / 112 |
| **4** | **20** | 2900.279 | 2967.316 | **-**1430.140 | **.84-.97** | **73 / 20 / 99 / 19** |
| 5 | 23 | 2906.279 | 2983.371 | -1430.139 | .58-.94 | 20 / 73 / 20 / 98 / 0 |

*Abbreviations:* AIC: Akaike Information Criterion, BIC: Bayesian Information Criterion, LCMM: Latent Class Mixed Modelling

**Figure 3:** Sensitivity analyses: Model estimated class-specific mean predicted trajectories of tobacco smoking with 95% confidence intervals.


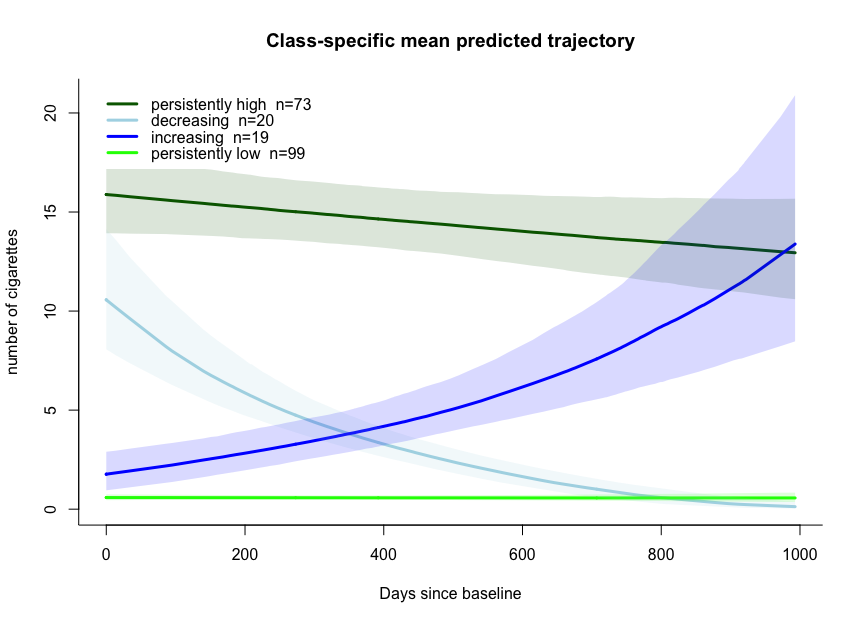

Supplement: Supplementary file 1 [file Data_Sheet_1.docx]
